# Therapeutic strategies of recurrent glioblastoma and its molecular pathways ‘Lock up the beast’

**DOI:** 10.3332/ecancer.2021.1176

**Published:** 2021-01-22

**Authors:** Shaimaa M El-khayat, Waleed O Arafat

**Affiliations:** 1 Cancer Management and Research Department, Medical Research Institute, Alexandria University, Alexandria 21568, Egypt; 2 Alexandria Clinical Oncology Department, Alexandria University, Alexandria 21568, Egypt

**Keywords:** glioblastoma, GBM, recurrent GBM, recurrent high-grade glioma

## Abstract

Glioblastoma multiforme (GBM) has a poor prognosis—despite aggressive primary treatment composed of surgery, radiotherapy and chemotherapy, median survival is still around 15 months. It starts to grow again after a year of treatment and eventually nothing is effective at this stage. Recurrent GBM is one of the most disappointing fields for researchers in which their efforts have gained no benefit for patients. They were directed for a long time towards understanding the molecular basis that leads to the development of GBM. It is now known that GBM is a heterogeneous disease and resistance comes mainly from the regrowth of malignant cells after eradicating specific clones by targeted treatment. Epidermal growth factor receptor, platelet derived growth factor receptor, vascular endothelial growth factor receptor are known to be highly active in primary and recurrent GBM through different underlying pathways, despite this bevacizumab is the only Food and Drug Administration (FDA) approved drug for recurrent GBM. Immunotherapy is another important promising modality of treatment of GBM, after proper understanding of the microenvironment of the tumour and overcoming the reasons that historically stigmatise GBM as an ‘immunologically cold tumour’. Radiotherapy can augment the effect of immunotherapy by different mechanisms. Also, dual immunotherapy which targets immune pathways at different stages and through different receptors further enhances immune stimulation against GBM. Delivery of pro-drugs to be activated at the tumour site and suicidal genes by gene therapy using different vectors shows promising results. Despite using neurotropic viral vectors specifically targeting glial cells (which are the cells of origin of GBM), no significant improvement of overall-survival has been seen as yet. Non-viral vectors ‘polymeric and non-polymeric’ show significant tumour shrinkage in pre-clinical trials and now at early-stage clinical trials. To this end, in this review, we aim to study the possible role of different molecular pathways that are involved in GBM’s recurrence, we will also review the most relevant and recent clinical experience with targeted treatments and immunotherapies. We will discuss trials utilised tyrosine receptor kinase inhibitors, immunotherapy and gene therapy in recurrent GBM pointing to the causes of potential disappointing preliminary results of some of them. Additionally, we are suggesting a possible future treatment based on recent successful clinical data that could alter the outcome for GBM patients.

## Introduction

A group of malignant brain tumours called ‘Gliomas’ arises from the supporting neuroglial cells (astrocytic or oligodendroglial cells). Although current WHO classification includes epigenetic, genetic and clinicopathological features of brain tumours, most clinicians still divide brain tumours into low-grade glioma including (Grades I, II) and high-grade glioma (HGG) (grades III, IV) [1]. Glioblastoma multiforme (GBM) WHO grade IV is the most aggressive and commonest of these brain tumours and constitutes up to 54% of all gliomas and 16% of all intracranial tumours (primary and metastatic) [2]. Despite researchers’ efforts to improve the outcome of patients with GBM, the only approved treatments are still maximum safe resection followed by radio-chemotherapy with alkylating agent temozolomide (TMZ) then adjuvant TMZ with tumour-treating fields [3], this initial stage of treatment takes around 9 months [4–6], and its result in 7–9 months progression-free survival (PFS) and 15 months overall survival (OS) [7]. However, 100% of GBM will recur and response to subsequent treatment is very minimal [8], that is why we need to understand the molecular basis that led to recurrence and possible targeted treatments that can be developed to overcome its resistance to almost all treatments in the recurrent setting.

**Figure 1.**
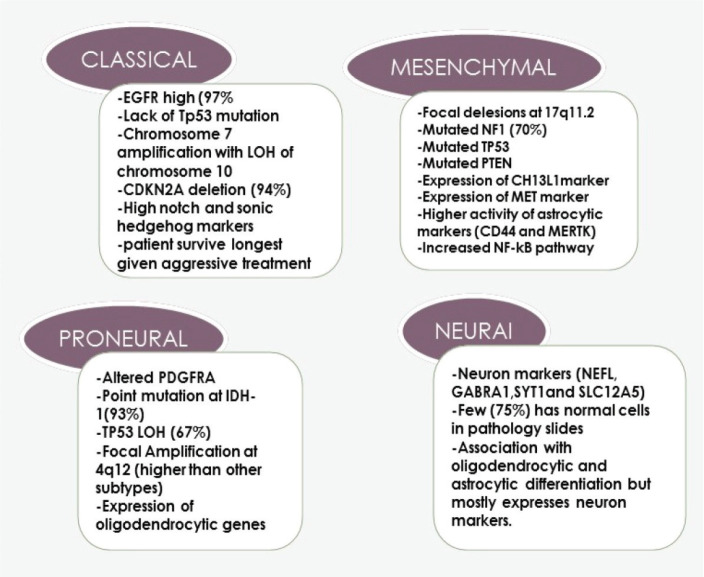
Four subtypes of GBM and the dominant genes and molecular abnormality in each group.

As suggested by the term ‘multiforme’, GBM is characterised by intra-tumour heterogeneity not only on cellular but also on molecular levels [9]. This heterogeneity is one of the principal reasons for therapeutic resistance and recurrence [9]. It is believed that this happens due to the biological selection of resistant malignant clones and then they acquire genetic alterations making them more aggressive after primary treatment [10]. One of the famous publications in which scientists extensively studied GBM at the molecular level was the cancer genome atlas (TCGA). TCGA has offered insights into the genetic regulation of GBM by a molecular GBM classification with the identification of molecular subgroups with prognostic significance [11, 12]. The four subgroups of GBM are classical, neural, pro-neural and mesenchymal as shown in Figure 1, pointing to the defective molecular pathway in each sub-group which could help to develop specific targeted treatments in the future and better designing of clinical trials on a molecular basis [12].

Till now, no clinical reflection of the four subgroups with only a slight survival advantage to the pro-neural subtype [13, 14].

This review article considers the major therapeutic strategies currently being investigated in the field of recurrent GBM, focusing on approaches with not only pre-clinical but also clinical data. We aim to discuss novel and experimental tyrosine kinase receptor inhibitors, immunotherapy and gene therapy pointing to the underlying pathways that lead to their promising role in recurrent GBM. We also added a final section on the most important future direction that scientists are trying to apply to treat recurrent GBM based on pre-clinical data to improve the outcome of these patients.

## Methods

We searched the MEDLINE, PubMed database for high impact factor journals at least 1.8 with high citations. We also searched clinical trials.gov for phase I, II trials with reported PFS, OS published on tyrosine receptor kinase inhibitors, immunotherapy and gene therapy in recurrent GBM and discussed them. Ongoing trials and pre-clinical relevant up-to-date studies related to this subject have also been discussed. We used in our search the keywords glioblastoma, GBM, recurrent GBM and recurrent high-grade glioma.

## Discussion

### Receptor tyrosine kinase (RTK)

The RTK inhibitors are one of the most extensively studied drugs in oncology, we will discuss in the following section four of the main growth factor receptors in GBM and targeted treatments against them focusing on the applied clinical experience from clinical trials.

#### Epidermal growth factor receptor (EGFR)

**Figure 2.**
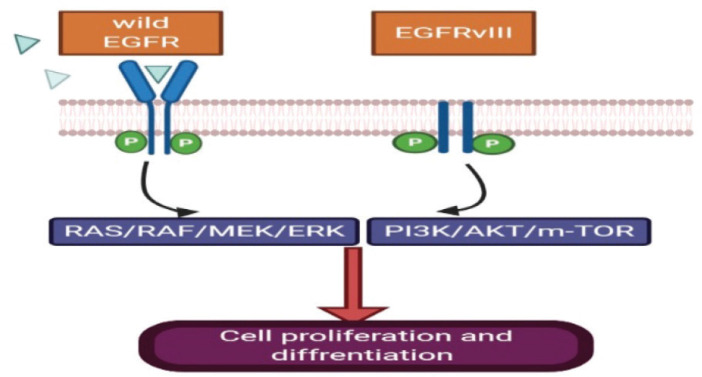
EGFRvIII has an extracellular domain truncation from exons 2 to 7, which results in the deletion of amino acids 6-273 and renders the mutant receptor incapable of binding the ligand. EGFRvIII can display constitutively active signalling independent of ligand.

Human EGFR (EGFR; HER-1) is over-expressed in 40%–60% of primary GBM tumours and mostly occurs in the classical subtype (Figure 1) [15], but EGFR mutation which leads to EGFRvIII expression (Figure 2) present in 20%–30% of primary GBM [15]. In this regard, a study of 186 pairs of primary and recurrent GBM samples found that patients with recurrent glioblastoma multiform (rGBM) do not represent specific molecular subtypes and almost 80% of recurrent diseases retain the same molecular abnormalities as in the primary tumour samples [16]. Therefore, scientists designed clinical trials investigating targeted treatment for recurrent GBM based on an almost similar percent of activating mutations in primary and recurrent samples. Although the EGFR gene is commonly amplified in GBM, this does not correlate with responsiveness to EGFR inhibitor in most of the trials. The mutations of EGFR in GBM is linked to ‘gain-of-function miss-sense mutations or in-frame deletions affecting the extracellular domain’ [17, 18]. EGFRvIII is always active regardless of the presence of ligand or not with dysregulated downstream pathways [19]. This mutant ligand-independent pathway is believed to create a state of ‘pathway addiction’ in which the tumour dies if debriefed from this signal by tyrosine kinase inhibitors (TKIs) [20].

EGFR receptor is activated by two mechanisms as mentioned above, either by receptor over-expression or multiple ligand-independent and ligand-dependent pathways which will lead to stimulation of subsequent downstream mitogen-activated protein kinase, phosphatidylinositol-3-OH kinase (PI3K) and Src kinase pathway besides signal transducers and activators of transcription (STAT) transcription factor activation [21]. These events starting from upstream mutation will lead to an intracellular cascade of events leading to gene transcription, cell proliferation, survival, invasion and angiogenesis (Figure 2).

Sometimes phosphatase and TENsin homolog (PTEN) activity is lost which mainly acts as a tumour suppressor protein that inhibits the PI3K pathway, in this situation, there will be resistance to EGFR kinase inhibitors [22]. Patients with PTEN-deficient tumours could benefit from downstream inhibition of the PI3K pathway, maybe at the level of the mammalian target of rapamycin (mTOR), with EGFR inhibitors.

EGFR TKIs are classified into three main categories: first-generation inhibitors that target EGFR and its co-receptor HER2 and bind to it reversibly like (gefitinib, erlotinib and lapatinib); second-generation irreversible inhibitors (afatinib, neratinib and dacomitinib) and third-generation TKIs.

Almost all trials for recurrent GBM patients ‘based on a high percentage of them expressing EGFR (40%–60%)’ evaluating EGFR TKIs utilised a continuous daily dosing schedule but also included unenriched participants (Table 1). Some studies evaluated TKI monotherapy, others evaluated the combination regimen.

Gefitinib was evaluated in phase II single-arm trial that included 57 patients during the first recurrence and found that no radiological response was found in them, PFS-6 (PFS at 6 months) was 13% and OS was 39.4 weeks. There was no correlation between EGFRvIII mutation’s presence or absence of EGFR over-expression with the outcome [23]. Another study that evaluated gefitinib in the neo-adjuvant setting found that its concentration at the tumour was 20 times more than that found in the plasma but this finding was not associated with downstream pathway inhibition. So the drug acts effectively on the EGFR receptor upwards, but no effect on downstream pathways, this is also observed with erlotinib [24] and lapatinib. These studies suggest that probably first generation EGFR TKIs do not inhibit the EGFR signalling in GBM effectively and the above-mentioned observation could be the reason for the failure of these drugs till now.

Erlotinib was tested on 44 recurrent GBM patients and again the radiological response was not observed and PFS-6 was 3% [25]. Later studies proved that erlotinib has poor central nervous system (CNS) penetration due to interaction with P-gp efflux transporter and breast cancer resistance protein [26]. Other study which compared erlotinib with chemotherapy at first recurrence found that the outcome was comparable in both arms and EGFRvIII mutation was linked to poor PFS [27].

Resulting from the hypothesis of thinking of the possibility that stimulation of downstream pathways or activation of other survival pathways may cause EGFR resistance, subsequent studies evaluated the combination of EGFR TKI with agents that inhibit these signalling pathways [28]. Many studies evaluated EGFR TKI combined with (mTOR) inhibitors that act as a mediator of the PI3K/AKT phosphatidylinositol-3 kinases/AKT, also known as protein kinase B (PKB), signalling pathway. Patients with recurrent GBM were evaluated in a phase I trial to determine the maximum tolerable dose (MTD) of gefitinib with (an oral mTOR inhibitor) and reported PFS-6 of 23.5% [29]. Then another phase II study which was a single-arm on 32 heavily pretreated, recurrent GBM patients evaluated combining erlotinib with sirolimus and found that no radiological response was found and PFS-6 was only 3.1% [30], and also, unfortunately, no correlation between OS and EGFRvIII, pEGFR and EGFR amplification.

Some of the finished and ongoing trials of anti-EGFR in rGBM are demonstrated in Table 1;

Depending on the pre-clinical findings that VEGF signal activation acts as a mediator of EGFR resistance, a combination of EGFR inhibitor and VEGF2R2 inhibitors was introduced to clinical trials to be tested [38, 39]. Phase II study evaluated erlotinib plus bevacizumab ‘a humanised monoclonal antibody against VEGF that is Food and Drug Administration (FDA)-approved for recurrent GBM’ [40] was conducted on patients with rGBM [41]. Erlotinib was administered at 200 mg/day and 500 mg/day for participants taking and not taking enzyme-inducing antiepileptic drugs, respectively, and bevacizumab was administered at 10 mg/kg biweekly. In the above study, PFS-6 was 28% and OS was 42 weeks, and these results were the same as that found with bevacizumab monotherapy.

As regards trials that evaluated second-generation EGFR, in phase I/II study of afatinib with or without protracted TMZ in patients with GBM after recurrence, provided that all patients received standard chemo-radiotherapy with TMZ at presentation [42]. The phase I results of the trial found that MTD of afatinib with TMZ is 40 mg/day for afatinib and 75 mg/m^2^/day for TMZ when combined. And the phase II results of the same trial found that the PFS-6 for the afatinib monotherapy arm, afatinib-protracted TMZ arm and combination arm was 3%, 23% and 10%, respectively. Different biomarkers were evaluated like EGFR, EGFRvIII, PTEN, pAKT and O6-methylguanine-DNAmethyltransferase (MGMT) but none of them correlated with the outcome although a non-significant association with EGFRvIII expression and better outcome were observed in patients treated with afatinib.

Dacomitinib single agent was evaluated in two phases II studies [43, 44]. The first one included two cohorts; one of them included patients with EGFR over-expression with no EGFRvIII mutation and achieved PFS6 of 13.3% and OS of 7.8 months, the second cohort included patients with EGFR over-expression and EGFRvIII mutation and PFS6 was 5.9% and OS of 6.7 months. The two cohorts received dacomitinib till disease progression or unacceptable side effects [35]. The second study contains three arms and still ongoing, one of the three arms is giving dacomitinib as a neoadjuvant treatment before surgery and this will help to determine the penetration of dacomitinib to the blood–brain barrier and also its capability of inhibiting intra-tumour phosphorylation. The other two arms include patients who are naïve and previously exposed to bevacizumab. Neratinib is also under evaluation in tumours with either EGFR mutation or amplification in phase II study [45].

#### Platelet derived growth factor receptor (PDGFR)

PDGFR is another member of the TKI family and is overexpressed in HGGs, especially in GBs [46]. Platelet derived growth factor receptor A (PDGFRA) is over-expressed in about 15% of GBMs [47]. That is why researchers make efforts to target this receptor and its pathway. Pre-clinical studies are undergoing to test PDGFR inhibitors *in vivo* and *in vitro* and some of these inhibitors are approved for clinical trials. Imatinib (Gleevec) is one of these drugs which has an inhibitory effect on PDGFR. Although imatinib has activity in other malignancies, it did not show significant activity in HGG especially GBM in the recurrent settings. The tumour growth and OS remained unchanged whether it was used as a single agent or in combination with hydroxyurea [48, 49]. Recently, *in vitro* studies on GB cells found that imatinib increases the migration and invasion of GB cells, a fact that explains the previous failures of the drug [50].

**Figure 3.**
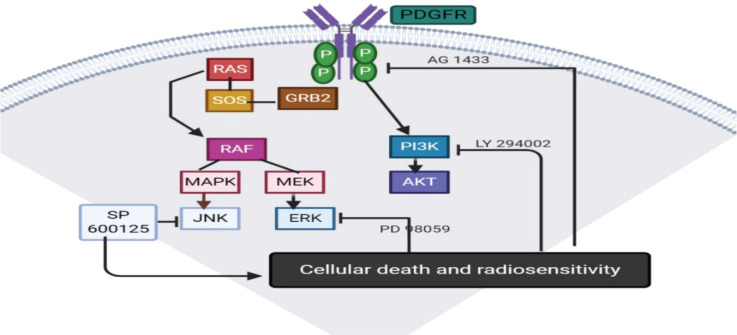
PDGFR pathway.

Tandutinib is another platelet derived growth factor receptor B (PDGFRB) inhibitor, which was evaluated in clinical trials in recurrent GBM and was found to have a little effect [51]. AG1433 is also another PDGFR inhibitory molecule that proved activity in pre-clinical trials in several *in vitro* HGG cell lines [6]. In 2019, it was tested on 11 and 15 HGG cell lines with radiotherapy or not, and found that AG1433 was effective and adding radiation to it does not increase its activity [52] (Figure 3).

#### Vascular endothelial growth factor receptor (VEGFR)

GBM is one of the highly vascularised tumours and increased microvasculature is one of its hallmarks of pathology [53]. Many previous studies focused on targeting angiogenesis which occurs by spurting of new capillaries and blood vessels and by also recruitment of endothelial cells to provide blood supply for the growing tumour [54]. VEGFR especially VEGFR2 is an important target in glioblastoma. Vatalanib (PTK787) is a VEGFR2, PDGFR and c-kit inhibitor, scientists found that it has a little effect on GBM alone with either chemotherapy or radiotherapy [55]. In phase II study, Sorafenib which is another VEGFR inhibitor with temsirolimus had a small effect on GBM [56]. Despite that tivozanib ‘an inhibitor of angiogenesis’ had good anti-angiogenic effects on GB, it failed to change the tumours’ volume [57]. Pazopanib also was combined with lapatinib but the results were disappointing [58]. Cediranib, ‘a small molecule inhibitor of VEGFR, PDGFR and c-kit’, showed a small improvement in the neurological status of the patients but did not change PFS or OS [59]. SU1498 is another VEGFR inhibitor that has limited activity on GBM [60].

Recent data suggest that YKL-40 is a good marker for angiogenesis in recurrent GBM for which targeted treatment may improve the outcome. YKL-40 is a mesenchymal marker which is named as ‘human cartilage glycoprotein-39 or chitinase-like protein-1’ and probably has an important role in migration and motility of glioma stem cells (GSCs) and their differentiation into endothelial cells, that is why it has a role in angiogenesis [61]. It was proven that YKL-40 causes up-regulation of VEGF expression and new tumour vasculature induced by YKL-40 is partially dependent on VEGF [62]; therefore, targeted treatments against YKL-40 could affect GB’s treatment.

#### Fibroblast growth factor receptor (FGFR)

Although FGFR mutations are not frequent in GBM, several studies suggest that modification of the FGFR signalling pathway stimulates GBM progression and patient survival [63]. Small molecules that inhibit the FGFR TKI are under investigation [64]. Some of them are non-specific to FGFR and act on other RTK like ‘lenvatinib, ponatinib, dovitinib and brivanib’, and others selectively target FGFR, like PD173074, BGJ398, AZ4547 and JNJ-493 [65]. A study, which used a large-scale shRNA to know the FGFR signalling to be targeted in glioma at the paediatric age, found that dovitinib, ponatinib, PD173074 and AZ4547 can inhibit the growth of glioma cells *in vitro* more than TMZ [66]. In December 2019, a trial involving BGJ398 in rGBM was completed, but no results have been published yet. Another phase I/II trial using TAS-120 is currently recruiting patients with metastatic solid tumours, regardless of fibroblast growth factor (FGF)/FGFR-related abnormalities [67].

### Multi-target tyrosine receptor kinase inhibitors (TRKI) agents

The multi-targeted approaches may represent a good choice for effective selection of resistant tumour subtypes. Vandetanib is one of these multitargeted TKI (VEGFR, EGFR) that was evaluated in clinical trials for patients with recurrent GBM. The drug was safe and tolerable but its antitumour effects were limited [68].

Another phase I clinical trial proved the safety of administrating vandetanib in combination with sirolimus in patients with rGBM [69]. Two other multitarget (cabozantinib) and (PD173074) are small molecules that act by inhibiting VEGFR and other receptors. Cabozantinib achieved good results *in vitro* and clinical trials, and PD173074 had good results *in vitro*. Sunitinib is a multi-kinase inhibitor of VEGFR, PDGFR, fms-related receptor tyrosine kinase 1 (FLT1), FLT1/kinase insert domain receptor (KDR), FLT3 and Ret Proto-Oncogene (RET) kinases with no encouraging results in patients with GBM [70].

### Immunotherapy in GBM

Immunotherapy stimulates the immune system to recognise, target and get rid of tumour cells. Many trials have focused on developing immunomodulating therapies to restore the functional ability of different immune cells against neoplastic cells and with promising results in several tumour types including melanoma, lung cancer, urothelial tumours and colon cancer [71]. Recent efforts and advances in translational research have led promising several strategies for immunomodulation in GBM, we discussed here the potential limitations and advances of immunotherapy in GBM. First limitations of immunotherapy in GBM is based on that immunotherapy has been most successful in tumours with high tumour mutational burden (TMB) but have yet to yield breakthroughs in GBM. GBM has low TMB despite its profound heterogeneity, rendering it an intrinsically immunologically quiet disease. Further suggested limitations of immunotherapy in GBM include the immunosuppressive environment that down regulates antigen presentation and disengages infiltrating immune cells [72, 73]. Also, immunotherapies can lead to inflammation within the intracranial space which could result in severe treatment-limiting neurological complications due to increased vasogenic oedema, autoimmune encephalitis and cytokine release syndrome [74, 75].

To leverage the natural immune response and restore its elimination ability of glioblastoma malignant cells, crucial understanding of the BBB and the tumour microenvironment and its complex interaction with the immune system is required In GBM, the BBB integrity is changed due to endothelial tight junctions damage reﬂecting molecular composition changes [76]. The BBB breakdown allows CD8 + T cells to migrate to the CNS, and stimulation of the innate and adaptive immune responses which produce cytokines and chemokines to recruit lymphocytes and up-regulate immune-modularity markers on T-cell surfaces [77]. Nowadays, it is also accepted that functional expansion secondary to high cerebrospinal ﬂuid (CSF) pressure [78]. Consequently, brain lymphatic vasculature provides an important pathway in both ﬂuid and immune cells circulation from CSF to systemic lymph system, suggesting a role in antigen presentation and immune surveillance of the CNS.

It is also believed that simultaneous ionising radiations’ exposure augments the strength of immunotherapy by: 1) a direct and indirect destruction to tumour cells causing cell deaths, 2) an alteration of the cancer stromal microenvironment and 3) activation of CD8 + T cells. Radiation induces activation of sequential biological mechanisms and biochemical events, including stimulation of interferon genes pathway and up-regulation of transforming growth factor β, leading to initiation of immune responses [79]. Therefore, co-administration of immunotherapy to block immune checkpoint is beneficial [80]. We will discuss here immune check point inhibitors and the dendritic cell (DC) vaccine as part of immunotherapy strategies in recurrent GBM. At the end, we will highlight genetic syndromes in paediatric GBM patients for whom immunotherapy could be the best choice for them based on recent clinical data.

### Immune checkpoint inhibitors (ICIs)

**Figure 4.**
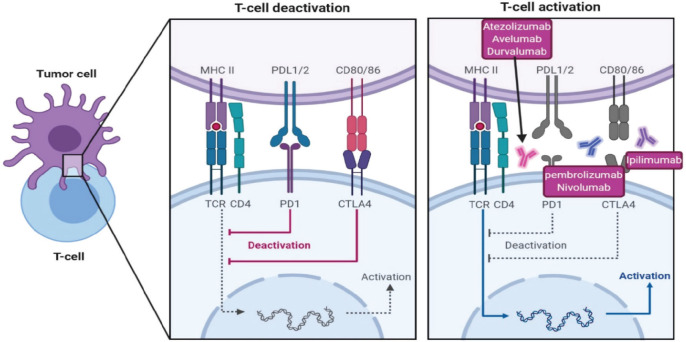
Immunotherapeutic pathways to in-activate T-cell against tumour cells and different immunotherapy to reactivate T-cells, CTLA4 inhibitor ipilimumab, PD1 inhibitor pembrolizumab and nivolumab and PDL-1 inhibitor atezolizumab, avelumab and durvalumab.

Cytotoxic T-lymphocyte-associated protein 4 (CTLA-4) and PD-1 pathways operate at distinct stages of an immune-response, resulting in negative effects on T-cell activity (Figure 4). CTLA-4 pathway is in the early stage of T cell activation, by binding of CD28 molecules on T-cells with B7-1 (CD80) and/or B7-2 (CD86) molecules on the surface of an antigen-presenting cell, mainly in the lymph nodes. On the other hand, PD-1 pathway blocks T cells at a later stage of the immune response through binding to PD-L1 and programmed death ligand 2 (PD-L2), in peripheral tissues [80] as in Figure 4.

Generally, anti-PD-1 drugs have a well-tolerated adverse event profile rather than anti-CTLA-4. It is suggested that a greater toxicity could be related to a better responses. Additional results are needed to decide whether this hypothesis is validated. Despite a different spectrum of adverse events, a safe and effective management of checkpoint inhibitors’ toxicity is mainly based on early sign and symptom recognition.

Overall, objective responses to ICI in rGBM were seen in the following trials shown in Table 2 the most important of them are as follows [81, 82].

In one retrospective analysis which investigated the effect of ipilimumab 3 mg/kg every 3 weeks with bevacizumab found an over-all response rate (ORR) of 31% in patients with rGBM [82]. A phase I trial found that the ORR of nivolumab single agent and nivolumab+ ipilimumab was 11% and 10%, respectively [83]. Other phase I study used atezolizumab (1,200 mg Q3W) in 16 patients with glioblastoma showed an ORR of 6.0% [84]. Three patients with isocitrate dehydrogenase 1 (IDH1)-mutant tumours had better PFS (5.5 months versus 1.2 months) and slight better OS (16.0 months versus 2.7 months) than patients who had IDH1-wild-type tumours. Some studies looked at the utilisation of immunotherapy as neoadjuvant treatment and one of them found that when pembrolizumab was given before surgery, patients had longer OS than patients who received it as adjuvant therapy only. Neoadjuvant therapy with pembrolizumab was linked to up-regulation of T cell and interferon-γ-related gene expression, and down regulation of genes related to cell cycle progression [85]. Similar changes were observed with neoadjuvant nivolumab in a phase II trial [86].

**Figure 5.**
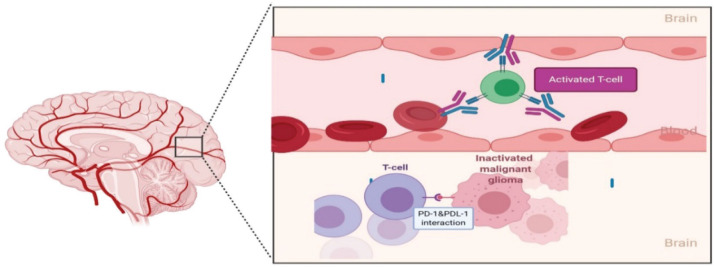
In recurrent disease, efficacy of nivolumab is limited by its inability to cross the BBB and a paucity of functional circulating T-cells with which to interact and form a protective barrier against subsequent possible PD-1/PD-L1 interactions. Exposed to numerous immunosuppressive influences within the glioma microenvironment, including uninhibited PD-1/PD-L1 interactions, T-cells already sequestered within the TME are expected to be heavily dysfunctional and unable to be rescued solely with immune checkpoint inhibition.

Ongoing, three important phase III studies investigating the role of nivolumab in newly-diagnosed and recurrent glioblastoma are awaiting the final analysis publication, but the initial results are disappointing, checkmate-143 was the only one included patients with recurrent GBM, the other two trials included primary GBM only. The first trial was a randomised, open-label, phase III CheckMate-143 trial (NCT02017717), nivolumab monotherapy did not show significant change of OS in comparison to bevacizumab in rGBM patients [87]. The second one combined nivolumab with radiotherapy in CheckMate-498 trial (NCT02617589) but also failed to significantly improve OS of patients with newly diagnosed MGMT-unmethylated glioblastoma, in comparison to chemo-radiotherapy with TMZ [88]. Lastly, in MGMT-methylated glioblastoma patients, adding nivolumab to the first-line standard of care chemo-radiotherapy with TMZ in CheckMate-548 (NCT02667587) did not show an effect on PFS which was one of the primary end points, and OS data is still pending [88]. Possible explanation of failure of PD-1 inhibitor nivolumab in checkmate 143 is attributed to impaired interaction between it and PD-1 receptors on patient’s lymphocyte due to either 1) physical barrier by BBB 2) Systemic lymphopenia and 3) Reduced T-cell expression of PD-1 receptors. Poor drug penetration of BBB has been linked to inability of drugs to reach glioma malignant cells. BBB does not allow particles larger than 400–600 Da to pass [89] and nivolumab has calculated molecular mass of 146 kDa [90]. PD-1/programmed death-ligand 1 (PDL-1) axis inhibition occurs outside tumour at lymphoid tissue peripherally then activated coated T-cells enter tumour microenvironment. In the recurrent setting, any activated T cells with PD-1 against the tumour are supposed to have been migrated to tumour site inside CNS where they are inaccessible to mono-clonal antibodies [91] (Figure 5). Another possible reason for failure of checkpoint inhibitors is that patients with GBM have heavily dys-functioning antigen-specific T-cells activation and usually permanently anergic towards tumour’s antigens. This is due to chronic antigen exposure which results in ‘exhausted T-cells’ and over-expression of PDL-1 tumour cells that their function may not be fully regained by PD-1 inhibitor [92, 93]. Single agent with anti-PD-1 is unlikely to reverse all factors that cause T-cell inhibition.

Lastly unlike adult brain GBM, paediatric GBM is linked to genetic syndromes like Li-Fraumeni (Tp53) syndrome and bi-allelic mismatch repair genes syndrome (bMMRD), it is associated with increased incidence of malignancies in the first years of life; most common malignancies include glioblastoma, haematological malignancies and gastrointestinal tract cancers. BMMRD results from homologous germline mutations in one of the following genes (PMS2, MLH1, MSH2 and MSH6). ‘DNA mismatch repair deficient (DMMRD) GBM’ has the highest mutational load in all cancers. Knowing that non-DMMRD cancers with high mutational load like melanoma, lung and bladder malignancies have high response to ICI lead to potential therapeutic opportunity to this subset of patients with GBM. One study did exome sequencing and neoantigen prediction on 37 dMMRD cancers (hyper-mutated) and compared them to adult and paediatric GBM patients, bMMRD GBM had high mutational load compared to sporadic paediatric and adult GBM. Based on this data, two siblings with dMMRD who had recurrent multifocal GBM treated with nivolumab and this treatment resulted in strong radiological response and significant prolongation of clinical response [94].

### DC vaccine

DC is a highly specialised antigen processing and presenting cell which plays a vital role in initiating immune response and useful in immunotherapy as providing a way for cytotoxic T lymphocytes, natural killer cells and cytokines to kill tumour cell directly or indirectly [95]. Several small clinical trials utilised DCs in GBM with conflicting results; some showed no clinical benefit, others showed significant durable response. Recent meta-analysis of six phase II randomised controlled trials [96] included 347 patients with recurrent or primary glioblastoma.

Patients who received DC vaccine had significantly prolonged OS (HR: 0.6995% confidence interval (CI): 0.49 to 0.97, *p* = 0.03) compared to the control group and a trend towards better PFS was also detected (HR: 0.76, 95% CI: 0.56 to 1.02, *p* = 0.07).

Moreover, the incidence of side effects was comparable between patients treated with dendritic cell vaccine and control (odds ratio = 1.52, 95% CI: 0.88 to 2.62, *p* = 0.14; I2= 0%) [96].

Some trials reported improvement from 13 month OS in the control group to 15.7–35.9 months OS in those patients who received DCs [95]. So data on this modality of treatment is still immature and results from phase III trials are urgently needed.

### Gene therapy in GBM

Gene therapy simply is delivering either tumour suppressor genes to the tumour to regulate its growth by inhibiting oncogenes, or delivering an inactive pro-drug to be activated at the site of the tumour to a lethal compound. GBM gene therapy until now has used different delivery vectors like viral vectors, non-polymeric NPs and polymeric NPs. We will discuss briefly these vectors in the following section [116].

#### Viral vectors

It is the first and commonly used vectors in GBM like neurotropic retroviruses and adenovirus that possess a specific ability to infect neuron and glial cells like, herpes simplex virus1 (HSV-1) [117, 118]. First trial in retrovirus evaluated HSV thymidine kinase (HSV-TK) which is the suicide gene in combination with ganciclovir (Cytovene) as the pro-drug. HSV-TK will convert the pro-drug ganciclovir to the active form ganciclovir triphosphate which inhibits DNA replication [119]. The results demonstrated limited transfection ability into the tumour [120].

Toca 511 is another retroviral vector which delivers tumour suppressor gene, cytosine deaminase (CD) and oral pro-drug Toca FC, CD enzyme converts 5-fluorocytosine to active 5-fluorouracil [121]. A phase I trial showed safety of the drug in patients with recurrent GBM with regression of the tumour at the infusion site and now this treatment is being evaluated in phase II/III trials [122].

As regards adenoviral vectors, a phase I trial evaluated adenoviral vector with wild P53 (Ad-p53) transfected into tumour cells showed minimal toxicity but limited ability to penetrate tumour tissue [123, 124]. Another phase Ib trial evaluated adenovirus V-tk with valaciclovir injected post-surgery to tumour bed with concomitant radiation followed by adjuvant TMZ in newly diagnosed patients with GBM [125]. It showed small survival advantage over standard treatment. However, CD3+ T cells increased after the new treatment, supporting immune-activation after this therapy. Then a phase II trial with the same treatment versus SOC demonstrated increase in OS from 13.5 months in SOC arm to 17.1 months with the new treatment [126].

Despite many studies of viral vectors in GBM, it resulted only in small survival advantage and the main challenge facing it is the ability to penetrate tumour tissue.

#### Non-viral vectors

Including polymeric and non-polymeric systems. Few non-polymeric vectors have been studied in GBM including liposomes, gold nanoparticles (NPs) and RNA NP. A transferrin receptor-targeted liposome vector SG-53 encapsulates P53 wild type plasmid DNA can cross BBB targeting glioblastoma cells leading to reduction in MGMT and can induce apoptosis in xenograft mice [127]. So, SGT-53 can increase chemo-sensitivity of TMZ and now under evaluation in phase II trial in combination with TMZ in patients with recurrent GBM [128].

Nu129, a spherical nucleic acid gold NP, contains siRNA targeting B-cell lymphoma 2 (BCL-2) like protein 12 which participates in tumour progression and resistance to apoptosis [129]. Nu129 has proved its ability to cross BBB and increases apoptosis in xenograft mice model and now is under evaluation in phase I trial in patients with recurrent GBM [129].

RNA NPs, for example, tested to deliver anti-miR-21 locked nucleic acid sequences to inhibit miR21 in xenograft GBM models in mice, with significant tumour regression [130]. RNA NP is considered to be a promising treatment although it is still in the pre-clinical phase [131].

### Future directions

Based on current evidence and preliminary results of clinical trials assessing new treatment strategies, clustering patients with recurrent GBM according to their molecular profile involved in their disease development will defiantly help optimising decisions in clinical scenarios. This fact can be attributed to the widely varying molecular nature of this disease and its unique micro-environment. The awaited clinical trials results mentioned in this review (Tables 1 and 2) and probably starting phase III trials for the most successful treatment strategies from phase I, II trials will definitely help approving new treatment strategies for these patients.

**Figure 6.**
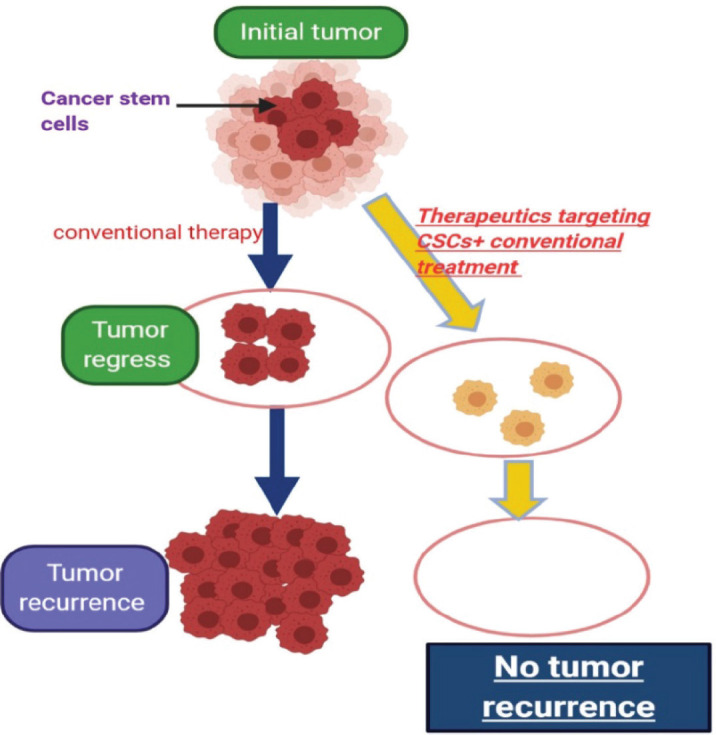
Describing the idea of targeting cancer stem cells in tumors which contain them.

Also, there is clearly much work to be done to identify novel therapeutic targets and to develop strategies for treating advanced thyroid cancer. Pre-clinical data suggests a number of areas that could be developed in the coming years. For example, GSCs is believed by scientists to be the main cause of relapse as it causes re-growth of the tumour after eradicating the main bulk of it by surgery and chemo radiotherapy [132] (Figure 6). It is important to understand the main pathways that lead to maintenance of GSCs and this area now represents an important scope of research in pre-clinical studies [133]. We mentioned here the most important pathways responsible for GSC maintenance ability, the ‘notch and the Wnt pathways’.

#### Notch signalling pathway

**Figure 7.**
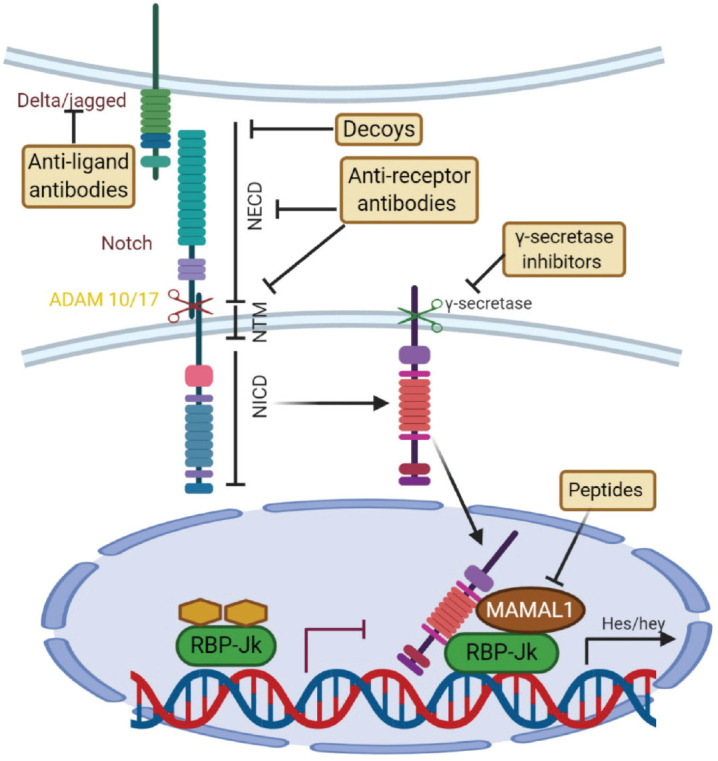
Canonical Notch signaling with points of intervention of current therapies. The interaction between Delta/Jagged-type ligands and Notch receptors leads to S2 cleavage on the extracellular site by “a disintegrin and metalloprotease” 10 (ADAM10) or ADAM17, which is followed by S3 cleavage by the γ-secretase–presenilin complex. The S3 cleavage gives rise to an intracellular Notch fragment (NICD) that translocates into the nucleus, where NICD binds to a protein complex containing recombination signal-binding protein Jκ (RBP-Jκ). This mediates the conversion of RBP-Jκ from a repressor to a transcriptional activator and is followed by the recruitment of the co-activator mastermind-like 1 (MAML1). These events lead to the de-repression of transcription of hairy/enhancer of split (Hes) and Hey. Several stages of the Notch signaling pathway are prone to pharmacological intervention and are labeled in the figure. Gamma-secretase inhibitors and blocking antibodies are already in clinical trials and decoys have been tested in animal models. Peptide inhibitors represent potential future treatment modalities. NECD, Notch extracellular domain; NTM, Notch transmembrane domain.

Notch pathway is important for determination of cell fate, proliferation, maintenance of cell quiescence, migration and regulate neural stem cell differentiation [134]. It starts by activation of γ-secretase by the jagged family ligands which leads to cleavage of notch receptors, then the intracellular notch receptor domain (NICD) translocates to the nucleus and formation of recombination signal binding protein for immunoglobulin kappa J region (RBPJ) and Mastermind-like 1 (MAML) complexes in the nucleus and activation of the hairy and enhancer of split (HES) and HEY genes that maintain multi-potency [135] (Figure 7).

CD 133-positive GSCs over-express genes like ID4 and FABP7 which are notch-pathway activator that leads to enhance infiltration ability of GBM [136]. Due to the above-mentioned function of notch pathway, inactivation of it may be effective for blocking GSCs and limiting sonic hedgehog (SHH/glioma associated oncogene (GLI) signalling pathway which plays an important role in oncogenesis especially neural progenitor regulation. That is why SHH/GLI is an important pathway for self-renewal and tumorigenicity of GSCs in which SHH/GLI pathway is active [137]. Studies found recently that SHH/GLI activity is important for Nanog regulation and expression which is a potent transcription factor and considered as a master regulator of many stem cells [138]. However, P53 decreases GLI activity which leads to Nanog down-regulation. So, P53 loss leads to Nanog up-regulation and maintaining stemness properties. Pre-clinical studies confirmed that this pathway contributes to GSC chemo-resistance and inhibition of SHH could potentiate activity of TMZ [139].

#### The Wnt/β-catenin signalling pathway

This pathway is important during CNS development and plays a role in self-renewal, differentiation and neural stem cell development [140]. However, aberrant activation of this pathway in the CNS leads to transformation into brain tumours. There is genetic and epigenetic factors which regulate the association between Wnt pathway and GSC maintenance [141]. Wnt 5A is another member of Wnt family which stimulates endothelial differentiation from GSC then neovascularisation that facilitates tumour growth and invasion [142]. In addition to above-mentioned mechanisms, Wnt signalling promotes (MGMT) expression which leads to TMZ resistance [143]. It is not possible to design a drug that targets Wnt pathway broadly as it is involved in many physiological processes inside brain and in other organs and this will lead to serious side effects. So developing strategies that target Wnt pathway at the tumour level is essential [140].

## Conclusion

Recurrent GBM is a fatal disease despite progression in understanding its molecular pathways and trying to target them. EGFR, PDGFR, FGFR and VEGFR inhibitors all showed few advantages although they are amplified or mutated in rGBM. YKL-40 targeting and multi-target of tyrosine receptor kinases may be beneficial. Immunotherapy has many limitations but it is still too early to decide that it is useless, especially DC vaccination which achieved significant advantage in some trials. A future direction towards targeting cancer stem cells pathways like notch and Wnt pathways could be an excellent solution for this disease.

## Conflicts of interest

The authors have no conflicts of interest.

## Funding statement

The authors have not received any funding for this work.

## Figures and Tables

**Table 1.** Some phase I, II trials of anti-EGFR in recurrent GBM and the status of each trial, whether finished or still ongoing.

Mechanism of action	Agent	Route of administration	Clinical trial	Trial phase	Status	citation
/	=	Oral	NCT01110876 ‘Vorinostat, Erlotinib and TMZ for recurrent GBM’NCT00301418 ‘Oral Tarceva Study for Recurrent/Residual GBM and Anaplastic Astrocytoma’	Phase I/II	Finished	[25, 31]
First generation EGFR TKI	Gefitinib	Oral	NCT00250887 ‘Pre- and Postoperative Use of ZD1839 (Iressa) in Recurrent GBM’	Phase II	Completed with no results yet	[32]
First generation EGFR/vascular endothelial growth factor receptor (VEGFR) TKI	AEE788	Oral		Phase I	Discontinued	[33]
Second generation EGFR/EGFRvIII TKI	Afatinib	Oral		Phases I, II	Finished	[34]
Second generation EGFR TKI	Dacomitinib	Oral	NCT01520870 ‘Safety and Efficacy of PF-299804 (Dacomitinib), a Pan-HER Irreversible Inhibitor, in Patients With Recurrent GBM With EGFR Amplification or Presence of EGFRvIII Mutation’ NCT01112527 ‘PF-00299804 in Adult Patients With Relapsed/Recurrent GBM’	Phase II	Finished	[35, 36]
Second generation EGFR TKI	Neratinib	Oral	NCT01953926 (SUMIT trial) ‘Neratinib HER Mutation Basket Study’	Phase II	Ongoing	[37]

**Table 2.** Most of the finished and ongoing immunotherapy trials in recurrent GBM.

Trial	Phase	Cohort	*N* of patient planned	Agent	PFS	OS
NCT03493932 [97]	I	Recurrent GBM	15	Nivolumab + BMS-986016	No results yet	
NCT02017717 [98]Checkmate 143	III	Recurrent GBM, newly diagnosed	A 626	Nivolumab Nivolumab + ipilimumab Bevacizumab	Final OS results not yet available	Median OS at 9.5 months nivolumab, 9.8 months (95% CI, 8.2–11.8); bevacizumab, 10.0 months
NCT02336165 [99]	II	Newly diagnosed or recurrent	159	First recurrence -durvalumab (10 mg/kg Q2W) as -durvalumab (10 mg/kg Q2W) + bevacizumab (10 mg/kg Q2W) -durvalumab (10 mg/kg Q2W) + bevacizumab (3 mg/kg Q2W).	/PFS-6 20% PFS > 7 weeks in 50%	OS-6 59%; OS12 44.4%/OS > 21 weeks in 36%
NCT02529072 [100] (AVERT)	I	First or second recurrence GIII, GIV glioma	6	Nivolumab+ DC vaccine	Primary end point safety and combination was as safe as Nivo single agent	
NCT02798406 [101] CAPTIVE/KEYNOTE-192	II	Glioblastoma or gliosarcoma tumour first time or recurrent	49	DNX-2401 (5e8 vp) delivered (adenovirus) intratumourally by cannula, followed by intravenous pembrolizumab every 3 weeks	Still recruiting	
NCT03058289 [102]	I, II	Refractory cancers	E 60	Experimental (Cohort A superficial tumours): INT230-6 low starting dose, low concentration per tumour. Experimental (Cohort B1 superficial or deep tumours): INT230-6 low starting dose, low concentration per tumour. Experimental (Cohort B2 superficial or deep tumours): INT230-6 medium starting dose, low drug concentration per tumour. Experimental (Cohort B3 superficial or deep tumours): INT230-6 high starting dose, low drug concentration per tumour. Experimental (Cohort C1 superficial or deep tumours): INT230-6 low starting dose, high drug concentration per tumour. Experimental (Cohort C2 superficial or deep tumours): INT230-6 medium starting dose, high drug concentration per tumour. Experimental (Cohort C3 superficial or deep tumours): INT230-6 high starting dose, high drug concentration per tumour. Experimental (Cohort D and E superficial or deep tumours): INT230-6 + anti-PD-1 antibodies	Still recruiting	
NCT02335918 [103]	I, II	Histologically diagnosed advanced non-small cell lung cancer, melanoma, colorectal, head and neck squamous cell carcinoma, ovarian cancer, GBM or renal cell carcinoma	A175	Varlilumab + Nivolumab	No results yet	
NCT02852655 [104]	I	Surgically accessible recurrent/progressive GBM	A 35	Pre-surgery MK-3475 Comparator: No MK-3475 at pre-surgery	Active not recruiting	
NCT02658981 [105]	I	Recurrent/progressive GBM	E 100	A1) Anti-LAG-3 Experimental: (A2) AntiCD137 (Urelumab) Experimental: (B1) Anti-LAG3 + Anti-PD-1 (nivolumab) Experimental: (B2) Anti-CD137 + Anti-PD-1 Experimental: (Intratumoural Studies) Patients pre-operatively receive drug from one of the four arms	Still recruiting	
NCT03233152 [106]	I	Primary and recurrent GBM	E 6	Ipilimumab + nivolumab	No results	
NCT02794883 [103]	II	Recurrent GBM	E 36	Tremelimumab Comparator: Durvalumab Comparator: Tremelimumab + Durvalumab	Recruiting	
NCT02311582 [107]	I/II	rGBM	E 58	Phase I: MK-3475 + MLA Experimental: Phase II: MK-3475 Only (Arm B) Experimental: Phase II: MK-3475 + MLA (Arm A)	Recruiting	
NCT02937844 [107]	I	rGBM	E 20	Anti-PD-L1 Chimeric Switch Receptor Engineered T cells	Recruiting	
NCT02866747 [107] (STERIMGLI)	I/II	Recurrent GBM	E 62	RT (Hypofractionated stereotactic radiation therapy (hFSRT) 24 Gy, 8 Gy per fraction) + Durvalumab 1,500 milligrams (mg) every 4 weeks Comparator: RT hFSRT 24 Gray (Gy), 8 Gy per fraction	Recruiting	
NCT02658279 [107]	N/A	Recurrent GBM with hyper-mutated subtype	E 44	Pembrolizumab	Recruiting	
NCT02829723 [108]	I/II	Advanced/metastatic solid tumours	E 151	BLZ945 BLZ945 + PDR001	Recruiting	
NCT02423343 [109]	I/II	Phase 1b, advanced refractory solid tumours in any line of therapy 2) Phase 2, recurrent or refractory NSCLC, or HCC with AFP ≥ 200 ng/mL	E 75	Galunisertib + Nivolumab (Phase 1b) Experimental: Galunisertib + Nivolumab (NSCLC) (Phase 2) Experimental: Galunisertib + Nivolumab (HCC) (Phase 2)	Recruiting	
NCT01375842 [110]	I	Recurrent GBM	16	Atezolizumab	1.2 month range (0.7–10.7)	4.2 (range 1.2–18.8+)
NCT02550249 [111]	II	Newly diagnosed and recurrent	29	Nivolumab	4.1	7.3
Retrospective [112]		Recurrent	37	NIVO (+ BEV)	4.6 (range 0.5–15.0)	6.5 (range 0.8–19.5)
Retrospective [113]	N/A	Recurrent GBM	16	Ipilimumab + bevacizumab	ORR 31%	
Retrospective [114]		Recurrent GBM	17 (10GBM)			OS 2.6 (0.4–11.6)
Retrospective [115]		Recurrent GBM	NIVO	16	2 (95% CI 1.3–2.7)	3.5 (95% CI 2.8–4.2)

LAG-3: lymphocyte activation gene-3; MLA: MRI-guided laser ablation; NSCLC: non-small cell lung cancer; HCC: hepatocellular carcinoma; AFP: alphafeto protein
